# Is Laser Photocoagulation Treatment Currently Useful in Diabetic Macular Edema?

**Published:** 2015

**Authors:** Pere Romero-Aroca

**Affiliations:** Ophthalmology Service, Hospital Universtari Sant Joan, Institut de Investigació Sanitaria Pere Virgili (IISPV), Reus, Spain

**Keywords:** Diabetic Macular Edema, Laser Photocoagulation, Anti-VEGF treatment

There has been a dramatic increase in the incidence of diabetes mellitus (DM) worldwide, which has been triggered by the growing obesity problem. In 1985, the World Health Organization (WHO) estimated that 30 million people worldwide had some form of diabetes; by 2000, the number had increased to 177 million.

Blindness as a secondary effect of diabetes mellitus is predominantly caused by diabetic macular edema (DME). By 2030, the incidence of DME is estimated to increase to 100 million (1). Although DME resolves spontaneously in approximately 35% of cases after six months (2), treatment regimens for the remaining 65% have become an important focus of study.

Diabetic macular edema treatments have changed in recent years after the introduction of intravitreal injections of anti-vascular endothelial growth factor (VEGF) (ranibizumab, bevacizumab, pegaptanib and the most-recent introduction of aflibercept), corticoids (triamcinolone or dexamethasone), and the sustained delivery fluocinolone acetonide vitreous inserts. All these new drugs are becoming more popular as the first line of treatments worldwide, but this also makes treatment more expensive. A combination of laser photocoagulation (LP) and intravitreal drugs is being investigated to find out whether such treatment might be just as effective and reduce costs, since LP alone seems to have lost popularity.

The lasers being used currently are solid lasers obtained from frequency-doubled diode or frequency-doubled Yttrium Aluminium Garnet (YAG), which emit at a wavelength of 532 nm, similar to those with the dry yellow-green to argon (514 nm) and the Krypton yellow (568 nm). Compared to the argon laser, the doubled diode or YAG lasers have a higher absorption in oxyhemoglobin (HbO) and hemoglobin (Hb), lesser dispersion (because of the long wavelength) and low absorption in xanthophyll. Other lasers, such as the 810 nm diode laser, are also being studied but will not be the focus of this present report.

*What Is the Current Place of Lasers in Treating DME?*

The laser has been the gold standard treatment option in comparative studies of intravitreal drugs used in DME treatments. Its effect was studied by the Early Treatment Diabetic Retinopathy Study (ETDRS) group during the 1990s and demonstrated that at 36 months:

• 65% of eyes of patients who had no laser treatment lost >15 letters on the Snellen chart,

• 33% of eyes of patients submitted for deferral LP lost >15 letters, and

• 13% of eyes of patients submitted to immediate laser treatment loss vision for >15 letters.

The study concluded that immediate laser treatment was effective in eyes with DME (3). From those results, LP became the treatment of choice, and all new treatments are being compared to those results. One important finding of the ETDRS group was the report on the effect of LP over time, showing that eyes of patients with clinically significant macular edema (CSME) increased in visual acuity (VA) by about 1% the first year, 6% at 24 months and achieved an improvement of 10% at 36 months following treatment. We can conclude that the laser was effective in treating DME and that the results were still relevant 36 months later. Thus, this timeframe of three years should be bared in mind whenever conclusions on whether a treatment is effective in patients with DME are made.

*Which Laser Technique Should We Use?*

The ETDRS group explained the two most important techniques of LP in patients with DME: the focal, for focal DME cases and the grid laser technique for the diffuse or more severe forms of DME.

They, furthermore, explained that a typical focal laser treatment entails mild, white burning beneath all of the leaking microaneurysms (MA) and other sites of focal leakage. A laser beam inflicts a burn raging in size from 50 to 100 µm by the duration for 0.05 to 0.1 seconds. It is important; however, not to burn within 500 µm of the center of the macula. A leaking MA is an area of retinal thickening on between 500 and 3000 µ from the center of the macula. A modified focal laser technique was introduced by Diabetic Retinopathy Clinical Research Network Group (DRCR.net group), which identified the same spot size (50 µm) and duration (0.05 to 0.1 seconds) to be incorporated into their guidelines, except direct whitening of the MS that was not required, and only a grayish lesion was recommended.

For grid treatment, the ETDRS suggested applying laser to diffuse leakages or nonperfusion within the following areas: 500 to 300 µm superiorly, nasally and inferiorly as well as 500 to 3500 µm temporally from the center of the macula. Attention should again be laid not to burn 500 µm within the center of the macula (4), respecting cells of the papillomacular bundle. The DRCR.net group (5) did include a minimal width apart two burns, as well.

*What Complications Should We Expect After Laser Treatment?*

Laser photocoagulation is not a safe technique as there are side effects possibly to occur after the burn. In particular, destruction of the retinal pigment epithelium may induce apoptosis of the surrounding retinal cells; moreover, those constituting macular area, what can ultimately affect VA. One of the most important effects that can reduce VA is the enlargement of a laser scar, referred to as atrophic creep, which represents a threat for eyesight prognosis if the laser has been applied too near the fovea. Schatz et al. (6) reported that enlarged laser scars reached the central fovea in eyes of 11 of 203 patients following grid LP for DME. Shah et al. (7) observed an expansion of laser scars in a subgroup of 18 patients at a 2-year follow-up visit, the average chorioretinal scar having expanded 50.1% per year in the first two years, and 4.6% per year thereafter.

*What Causes the Expansion of Laser Scars?*

One can speculate that because of their density, same spot size of LP destroyes more photoreceptors in the posterior pole than in the mid periphery. Furthermore, the photoreceptors cross-talk with each other through horizontal or amacrine cells, so necrosis of regional photoreceptors might lead to apoptosis of surrounding cells, possibly causing laser scars to gradually expand at a higher rate in the posterior pole. This expansion rate was even higher four years after treatment and lasers with lengthier wavelengths contribute to larger areas of chorioretinal atrophy in comparison to lasers with shorter wavelengths.

*Are There any Other Complications of Laser?*

If the laser burn affects the Bruch’s membrane, a choroidal neovascular membrane can grow underneath the neurosensory retina in the burn scar. This serious complication might be due to the use of repeated, small-size, short-duration lasers or intense laser burns, or both. These membranes can enlarge and reduce VA secondary to destruction of the retina, luckily, they respond well to intravitreal anti-VEGF agents (8). Finally, other secondary effects generally involve retina, such as photophobia or the appearance of scotomas in the visual field.

*So, What Place Does The Laser Currently Have in DME Treatment?*

The Royal College of Ophthalmologists (RCO) describes that: “overall, photocoagulation reduces the risk of moderate visual loss (defined as a doubling of the visual angle, equivalent to a loss of about two Snellen lines) by 50-70%. However, it is clear that photocoagulation therapy does not lead to an improvement in vision per se and while it reduces the risk of visual loss in the majority of patients, particularly those with PDR [proliferative diabetic retinopathy (PDR)], a significant proportion do not benefit from photocoagulation, especially for macular disease”. The RCO assigned an Evidence Level 1 to macular LP (focal or grid laser) when VA is reduced to 20/32 or less (9).

All studies of diabetic macular LP were referenced in the large ETDRS series study, but this study had some limitations based on the description of the treated DME types. For example, the ETDRS did not compare their laser treatment parameters to other therapies for DME, nor did the macular laser treatment described by ETDRS differentiate between focal and grid laser treatments. Finally, the treated lesions were identified using fluorescein angiography despite macular LP being the gold standard for DME treatment.

In the 21st century, the principal treatment indication for LP is for those edemas that affect the macular region without involvement of the CSME. Some patients who have lesions in CSME and low VA also have DME without a predominant MA as the focus. In these cases, some clinicians prefer intravitreal injections as first-line therapy and others favor the grid laser.

Generally, the grid laser seems to be used only in cases of DME that are unresponsive to intravitreal drugs. In these chronic cases that have difficulty responding to anti-VEGF treatment and corticoids, the grid laser seems to be of benefit, but the small improvement achieved in most cases makes it a poor option in the treatment of DME.
